# Edinburgh Lamarckians? The Authorship of Three Anonymous Papers (1826–1829)

**DOI:** 10.1007/s10739-021-09646-5

**Published:** 2021-08-20

**Authors:** Pietro Corsi

**Affiliations:** University of Oxford, Oxford, UK

**Keywords:** Evolution, Periodicals, Jean-Baptiste Lamarck, Étienne Geoffroy Saint-Hilaire, Robert Jameson, André d’Audebard de Férussac, Alexandre Bertrand

## Abstract

In the space of four years, from 1826 to 1829, the *Edinburgh New Philosophical Journal* published three anonymous articles seemingly advocating doctrines inspired by Jean-Baptiste Lamarck. Decades of scholarship have initially attributed the most outspoken of the three articles, the 1826 “Observations on the Nature and Importance of Geology,” to Robert Grant, and subsequently to Robert Jameson, thanks to a critical reassessment by James Secord (1991). More recently, scholars have also ascribed to Jameson an article published in 1829, “Of the Continuity of the Animal Kingdom by Means of Generation from the First Ages of the World to the Present Times.” A third short contribution, the 1827 “Of the Changes which Life has Experienced on the Globe” has been credited to the Franco-German Ami Boué. Research undertaken over several years has led to the identification of the three authors hiding behind the veil of anonymity. They were not the ones scholars have agreed upon, nor were they really “Lamarckians.” The discussion of the ways in which the three texts reached Edinburgh broadens our understanding of the daily working practices of contemporary periodicals and of the networks of circulation of texts at the Continental level. Finally, when considered within their proper conceptual and social context, the three articles throw light on the many ways in which, during the 1820s, European amateurs, naturalists, and journalists debated the succession of life forms throughout the history of the Earth.

Over the last few years, students of early nineteenth century British evolutionism have repeatedly brought to the forefront the question of the authorship of three articles published in the *Edinburgh New Philosophical Journal* in the space of four years, 1826 to 1829. Of these, “Observations on the Nature and Importance of Geology,” published in October 1826, had already been at the center of attention for several decades (Anon. 1826). The article has traditionally been seen as the first public endorsement in the British Isles of a Lamarckian interpretation of the succession of fauna and flora throughout the ages of the Earth. Discussion of the authorship of this contribution has more recently been paired with hypotheses concerning the authorship of two further anonymous articles, “Of the Changes which Life has Experienced on the Globe,” a short text published in 1827 (Anon. 1827a), and “Of the Continuity of the Animal Kingdom by Means of Generation from the First Ages of the World to the Present Times,” which appeared in the April 1829 instalment of the *Edinburgh New Philosophical Journal* (Anon. 1829b).

The 1826 article showed sympathy for, and knowledge of, Lamarck’s doctrine of the transformation of species, although the author refrained from fully endorsing what he considered the flights of imagination the French naturalist indulged in (Anon. 1826, pp. 296–297). Contrary to many simplifications of his colleague’s theory then current, the anonymous writer did not ascribe to Lamarck the view that life developed from monad to man in a single line of descent, an interpretation Lyell—among many others—favored a few years later. The anonymous author rightly pointed out that, according to Lamarck, there had been at least two separate lines of development, one starting with infusoria and one originating in parasitic organisms the French naturalist generically called “worms.” Worms spontaneously generated within already existing organisms, learned to live in the outside world, and went through a series of adaptative changes, leading to the appearance of vertebrates and ultimately of mankind (Anon. 1826, p. 296). True to its title, “Observations on the Nature and Importance of Geology,” the 1826 article was ostensibly devoted to extolling the worth of geology, a new discipline drawing from all departments of physical investigation and capable of important useful applications, especially to agriculture.

The 1827 short article “Of the Changes which Life has Experienced on the Globe” assumed that the history of life on Earth had been subject to constant and major changes due to the dispersal of the primordial high internal heat, decrease in volcanic activity, the lowering of the sea levels, and the general cooling of climatic conditions. Primitive life was essentially the same from the poles to the equator. Physical changes occurring at the surface of the Earth, produced by the progressive decrease in temperature, forced many forms of life to migrate towards the Tropics and from mountains to plains, as witnessed by the many organisms once living in northern latitudes now to be found only near the equator. Many species did not manage to adapt or move and became extinct; “new species appeared with new conditions of existence” (Anon. 1827a, p. 300). Mankind had appeared only when a state of climatic equilibrium was established and geoclimatic change had ceased or had become almost “imperceptible.” The article ended with a critique of catastrophism and a declaration of faith in the “laws of order and permanency which rule the universe” (Anon. 1827a, p. 301).

The third article (April 1829) offered a sympathetic précis of a memoir Étienne Geoffroy Saint-Hilaire (1772–1844) read at the Académie des sciences on 23 March 1829, in which he had maintained that fossil animals were the ancestors of living ones. After eulogizing Lamarck and his innovative work on the action of external circumstances on organisms, Geoffroy asserted that directional variations in the composition of the atmosphere had been mainly responsible for organic change, an implicit reference to relevant recent publications by Adolphe Brongniart (1801–1876), discussed below. It should be noted that the tribute to Lamarck was qualified by the remark that, although the latter’s work displayed the marks of a genius ahead of his time, most of the evidence he actually provided for his views was “far from being perfectly correct” (Anon. 1829b, p. 154). The article ended with the announcement by Geoffroy that his experiments on chicken eggs carried out at an incubation farm at Aulnay, on the outskirts of Paris, were producing encouraging results: the interference with the physical conditions of the incubation produced marked differences in the chicks. He was now free to spell out for the first time the full philosophical implications of such experiments, which he could not do “at a period when science was under persecution” (Anon. 1829b, p. 155).

The 1826 article has traditionally been attributed to Robert Edmund Grant (1793–1874), the so-called Edinburgh Lamarckian. Adrian Desmond supported the attribution, albeit cautiously, in articles he published in the early 1980s and in his groundbreaking, influential *The Politics of Evolution* (Desmond 1984, 1982–1984, 1989). In 1991, James Secord disrupted the consensus and powerfully argued that serious doubts could be raised against the attribution to Grant.^1^ He proposed instead that Robert Jameson, the professor of natural history Darwin found so unbearably boring during his days in Edinburgh, was hiding behind the veil of anonymity. Jameson was then the editor of the *Edinburgh New Philosophical Journal* and well known in the British Isles and in Europe as a leading mineralogist and follower of the German star of the discipline, Abraham Gottlob Werner (1749–1817). Despite Secord’s predominantly cautious approach, the view that Jameson was a closet evolutionist and the author of the 1826 article is today widely shared and has even found its way into the Wikipedia entry devoted to the Scottish naturalist (Ruse ([1996] 2009, p. 103; Eldredge 2015, pp. 47–48).^2^

Secord’s conclusion provided the starting point for the work Bill Jenkins has been devoting since the early 2000s to the “Edinburgh Lamarckians”: Jameson was not alone in his beliefs, Jenkins argued. He was only the most prominent figure within a group of naturalists sympathetic to Lamarck, to Geoffroy Saint-Hilaire, and to the transmutation of species. Jenkins’s PhD thesis (2015), now revised as a monograph, *Evolution Before Darwin: Theories of the Transmutation of Species in Edinburgh, 1804–1834* (2019), attempted to reconstruct the careers and standpoints of physicians and naturalists who showed marked sympathy for broadly Lamarckian tenets and, during the late 1820s and 1830s, for Geoffroy Saint-Hilaire’s (Lamarck’s alleged supporter) geo-climatic directionalism.^3^ In his thesis, Jenkins attributed to Jameson the 1826 and the 1829 articles for which Desmond had proposed Grant, but not the 1827 one, “Of the Changes which Life has Experienced on the Globe,” because the anonymous author seemed to follow a Plutonist interpretation of the origin of the Earth, which the Neptunist-Wernerian Jameson strongly opposed.

I was not convinced that Jameson authored the 1826 article and formed the impression that the article was due to a German-speaking author deeply versed in Wernerian natural history. The fact that textual traces of the 1826 article could be found, as Secord had pointed out, in the short preface the Edinburgh professor wrote for the fifth edition of Cuvier’s *Essay on the Theory of the Earth*, published in 1827, could indicate that Jameson had lifted a passage from a source he knew well, that is, the article he had published in his journal rather than that he authored both.^4^ In view of Jameson’s standing in Edinburgh, and of his familiarity with the European natural history scene from the beginning of the century, I found it difficult to explain why Jameson never said a word on beliefs he could have expressed in many different ways, with all the caution his position and respectability required—if, indeed, the views expressed in the articles under review were as dangerous and unguarded as some historians have assumed. After all, during the 1820s Jameson’s friend John Fleming had spoken with respect of Lamarck without incurring criticism (Fleming 1822, p. 14; 1829, p. 320). Moreover, since Jameson was the editor of the journal and a rather domineering personality at that, readers—at least in Edinburgh—could assume, wrongly or rightly, that he was not so opposed to doctrines articulated in a volume bearing his name on the title page. So, if he had avoided making his true beliefs known in order to preclude opposition on scientific or religious grounds, why did he print them behind the veil of anonymity in a journal of which he was the well-known editor? More fundamentally, as we are going to consider below, were the views expressed in the articles under consideration truly subversive and dangerous, let alone truly Lamarckian? If not, then Jameson could have safely hosted them in his journal, which reinforces the point that he could have signed the articles after all.

In the first section of this paper, I will identify the authors and the Continental sources for the three articles, following the chronology of their discovery. The search strategies adopted had to be changed over a period of two decades. A solution came only when online resources covering nineteenth century natural history publishing at Continental level reached critical mass. The second section will detail the available evidence on how and why the original texts reached Edinburgh, and reveal the almost daily exchanges of texts and information between Edinburgh and Paris. Finally, I will examine the contents of the articles within the context of their author’s careers and theoretical allegiances, not through the reductive and to some extent distorting lenses of Lamarckism. New evidence will emerge on the relationship between politics and science in Restoration France, especially during the second half of the 1820s, when opposition to the increasingly harsh predominance of ultra-royalist factions within government determined the formation of short-lived, broad alliances involving moderate royalists, nostalgic admirers of Emperor Napoleon, and even moderate republicans.

## Beyond Anonymity

### 1829: Geoffroy Saint-Hilaire at the Académie

Several years of searching for the author of the 1826 article on the importance of geology proved fruitless, in spite of successive attempts, repeated over the years, at perusing as many German journals and books as possible, looking for a title or a text deploying similar arguments. I was unexpectedly luckier with the third anonymous article, the 1829 summary of Geoffroy’s theories. Reading the article again a few years ago, it reminded me of a text I had annotated when researching my paper on the complex relationship between Lamarck and Geoffroy (Corsi 2011). A line-by-line comparison of the two texts established that the 1829 article was the translation of a summary of a memoir Geoffroy had read at the Académie des sciences on March 23, 1829. It was published in the opposition paper *Le Globe*, which from 1824 to July 1830 expressed the views of several political and intellectual factions opposed to the extreme right-wing policies of Charles X and his governments. As Pierre Leroux (1797–1871), one of the founders of the journal and its managing editor (“gérant”), recollected a few years later, *Le Globe* was “the arch that contained Doctrinaires, Eclectics, Liberals, Jacobins, from which Socialism too emerged” (Viard 2009, p. 49).^5^ Leroux had attended Geoffroy’s lectures and was on good personal terms with the naturalist. From 1824 until its demise in 1831, *Le Globe* paid consistent and sympathetic attention to Geoffroy’s interventions on the floor of the Académie. Due to Cuvier’s prominent role in key branches of government, such as the Council of State, support for his vociferous opponent Geoffroy assumed a political dimension. The restrictive legislation on the press and censorship forbade *Le Globe* to comment on political subjects. Thus, taking a side on “philosophical” issues in natural history—favoring, for instance, the unity of organic composition or the mutability of species—was seen by contemporaries as an implicit indictment of Cuvier, a well-known prestigious member of the administration, whom Geoffroy openly accused of hampering the progress of science to suit his conservatism.^6^

The actual author of the 1829 article on Geoffroy and the succession of life forms throughout the ages of the Earth was, however, Alexandre Bertrand (1795–1831). Bertrand and Leroux had met when attending secondary school in Rennes. They were staunch opponents of the Restoration and joined the Carbonari secret society (on their way to Saint-Simonianism) after failing to graduate from the École Polytechnique. When *Le Globe* was established, Bertrand served as a sort of science editor to the journal, writing weekly reports on the meetings of the Académie des sciences and the Académie de médecine. In 1827, Cuvier famously tried to stop Bertrand from attending the sessions; he succeeded in obtaining a measure of restriction that was never implemented thanks to the opposition of Jean Baptiste Joseph Fourier (1768–1830), perpetual secretary for the mathematics section from 1822, Geoffroy, and André-Marie Ampère (1775–1836) (Leroux^1836^, p. 643; Goblot 1995, p. 78; Csiszar 2018, pp. 79–82). Fourier made use of Bertrand as his personal copyeditor and ghostwriter.

Bertrand’s rather surprising personal stand on the question of transformism will be considered in the last section of this paper. It is appropriate to point out at this stage that he, too, like Leroux, admired Geoffroy, though he expressed reservations on the latter’s emphatic and at times over the line style (Bertrand 1828a, p. 379).^7^ He had attended Geoffroy’s lectures at the Muséum and assiduously reported on the anatomist’s exploits when addressing the Académie. The subject matter of the 1829 paper concerned (as we will see below) a memoir by François Désiré Roulin (1796–1874) on the changes domesticated European species underwent when finding freedom in the South American wilderness (Roulin 1828). Roulin had just returned from a long journey to Colombia (1822–1828), undertaken at the suggestion of Alexander von Humboldt and Jean-Baptiste Boussingault (1801–1887) (Viard 2009, p. 58).^8^ The fact that Roulin had married the sister of Bertrand’s wife, and had known both Bertrand and Leroux since their childhood days in Rennes, helps explain why the first memoir he ever presented to the Académie, interesting though it was, was thought worthy of a very long summary by Bertrand (Roulin 1828; Combes 1929).^9^ Geoffroy, by then a good friend of Leroux and Bertrand, managed to be charged with the task of reporting on Roulin’s memoir, together with Étienne-Renaud-Augustin Serres (1786–1868), Geoffroy’s pupil and friend, though it was the latter who wrote and read the report.

As will become clear in the following pages, the solution of the authorship puzzle for the three articles under discussion has raised a host of new questions for which answers are not simple nor readily available. Concerning the case at hand, if there is no doubt that the article published in the *Edinburgh New Philosophical Journal* was a translation of Bertrand’s contribution to *Le Globe*, several baffling matters remained to be accounted for. The very dating of the original French publication posed problems. Geoffroy’s full-length memoir was printed in volume 17 of the *Mémoires du Muséum national d’histoire naturelle* bearing the date 1828, whereas the summary provided by Bertrand on March 23, 1829, stated that the text had just been completed and read to the Académie on that date. This was the text translated into English. Since it would be tedious to retrace all the steps that have helped in establishing the actual chronology of events, I will only provide a synthetic account of the final result.

Roulin had personally read his memoir on South America on September 28, 1828, and Bertrand published a lengthy summary of it in the October 6 issue of *Le Globe*. Geoffroy had read his report on December 8, 1828, and Bertrand duly noticed it in *Le Globe* on December 13, 1828. After discussing the importance of Roulin’s work for understanding the process of domestication and praising the work of his own son Isidore (which Geoffroy rarely failed to do) on the varieties of domesticated mammals, the rapporteur “engaged in considerations of a very high order concerning the changes that races and species may have undergone in times prior to our own, when the influence of external circumstances was more powerful, and acted more broadly” (Bertrand 1828b). According to Geoffroy himself, writing in March 1829, the transition from domestication to speciation had occurred to him on the spur of the moment: “My mind being pre-occupied with old ideas respecting the antediluvian animals, there escaped me, in drawing up my report, a reflection which, to be rightly apprehended, would have required a greater development. This has been remarked, and I have been enjoined to do justice to the subject” (Anon. 1829b, p. 153; Bertrand 1829, p. 207).^10^

In this typical example of his contorted style, in March 1829, Geoffroy told his readers that in December 1828 colleagues at the Académie had objected to his “high order” remarks. The action of external circumstances on domesticated species transplanted to South America, they seemingly argued, did not warrant the conclusion that fossils were the ancestors of living organisms. Thus, the memoir Geoffroy started working on in early December 1828, which he announced to the Académie as completed on March 16, 1829 and read on March 23, was written in answer to a direct, pressing challenge. Knowing the irony Cuvier bestowed upon Geoffroy’s claim that his experiments on chicken’s eggs were providing a model for the succession of life forms throughout the history of the Earth, one may hypothesize that the anonymous and generic “colleagues” referred to as pressing for a full theoretical statement spoke with the voice of Geoffroy’s formidable antagonist (Corsi 1988, pp. 256–257).

A final puzzle needed to be solved: how could a memoir completed in March 1829 be printed in the *Mémoires du Muséum* volume bearing the date 1828? Archival work established that volume 17, 1828 of the *Mémoires* was distributed to the professors of the Muséum, the first recipients of the early copies, only in late April 1829: it had therefore been printed after March 23, 1829. So, Bertrand’s summary in *Le Globe* was the first public announcement of the thesis Geoffroy had put forward linking extinct and living organisms. It is only the irregular appearance of the *Mémoires*—as of many similar periodicals—that gives the impression that Geoffroy’s full text had been read and printed at the end of 1828 (Corsi 2016).

### 1826. “Observations on the Nature and Importance of Geology”

A few years ago, when providing a summary of the findings outlined above, I called attention to the presence of German-speaking naturalists in Edinburgh since the end of the Napoleonic wars (Corsi 2016). I mentioned the then unfairly neglected Ami Boué (1794–1881), a Hamburg-born geologist, an 1817 graduate of the Edinburgh medical school and a socialite who made it his business to know everybody in Europe. Boué lived several years in Paris and was among the founders of the French Geological Society in 1830 (Boué 1879).^11^ He was a transformist of some sort, although it would be very reductive if not wrong to consider him a “Lamarckian” in spite of the admiration he expressed towards the French naturalist. Thus, for instance, in 1834 Boué expressed the conviction that in former times a primeval vital power could have spontaneously generated complex organisms, a possibility Lamarck had firmly excluded. Boué also believed, against Lamarck, that among the simplest organisms there were many intermediaries between plants and animals (Boué 1834, pp. 115–116). I referred to Boué as a possible candidate for the authorship of the 1826 article on the progress of geology, but rather than claiming that this was the case, I simply wished to call attention to the several German-speaking naturalists who had studied in Edinburgh under or with Jameson and kept in touch, some of whom had shown an interest in discussing transformism (Corsi 2016, pp. 118–119; 2011). I also mentioned the Austrian Wilhelm von Haidinger (1795–1871), who became in 1849 the first director of the Kaiserlich-Königliche geologische Reichs-Anstalt in Wien; I could also have cited the Swiss, French speaking Auguste Verdeil (1793–1856), Boué’s fellow student in Edinburgh, who in 1818 took a medical degree with a thesis on the relationship between geology and health, *Dissertatio physica inauguralis de situs geologici efficacia in vitam animalem*, dedicated to Robert Jameson. These former pupils and colleagues were providing Jameson with first-hand information on trends and debates attracting attention on the Continent. Though seemingly promising, the line of investigation centered on Boué or on other German-speaking naturalists who had lived in Edinburgh in the 1810s and 1820s did not bring the hoped-for results.

As mentioned above, the cracking of the authorship of the 1826 article has taken several years. Encouraged by the success with the 1829 Geoffroy article, it seemed worth renewing my efforts. A slight change of strategy involved translating from English into German sentences or strings of words I considered (or hoped to be) specific to the text in question. I then ran these excerpts through all the online archives I was familiar with. The wealth of books now available online in key German and Swiss libraries brought an almost immediate result. The 1826 article translated into English the opening section of Albrecht Rengger’s (1764–1835) *Beyträge zur Geognosie: besonders zu derjenigen der Schweiz und ihrer Umgebungen* (Rengger 1824). As Koen Tanghe pointed out to me, the translator took some stylistic liberties with the original text, as was often the case with all translations at the time.^12^ A long footnote quoting the closing sentences to Cuvier’s “Discours préliminaire” to the *Recherches sur les ossemens fossils* was also suppressed, but on the whole the English language text faithfully rendered the sequence of paragraphs and the arguments deployed (Cuvier 1812, pp. 115–116; Rengger 1824, pp. 14–15).

The *Beyträge* was in fact a collection of essays, all but one by Rengger, dedicated to Hans Conrad Escher von der Linth (1767–1823), a former high-ranking Swiss politician and a close friend of the editor. The volume published in 1824 was the first of a projected series. Financial problems involving the famous publisher Johann Friedrich Cotta (1764–1832), and the latter’s death in 1832, stopped the publication of volume two, ready in 1829. The opening text on the importance of geology was not signed and only displayed the indication of a place and date of composition, “Lausanne 1822,” different from the ones stated in the short preface to the volume, signed by Rengger, “Aarau in Maymonat, 1823.” There appeared to be room to doubt that Rengger was the author of the introductory essay. Letters by Rengger to his friend Escher helped confirm that the editor was also the author of the opening section, composed in 1822, when he was living in Lausanne (Wydler 1847, vol. 2, pp. 307–309).

Rengger is at first sight a very implausible author of an alleged radical and groundbreaking scientific article, often seen by historians as the first full expression of Edinburgh Lamarckism. He had been a well-known protagonist of Swiss political life, serving as Minister of the Interior of the Confederation who sat at the Congress of Wien. Before 1815, he was the able negotiator of conditions aimed at limiting the ravages French troops were inflicting to parts of the Confederation (De la Harpe 1836; Flach 1898). He is probably better known as the editor of his nephew—almost an adopted son—Johann Rudolph Rengger’s (1795–1832) *Reise nach Paraguay* (Rengger 1835). Johann Rudolph had acquired European fame for the book he wrote together with Marcelin Longchamp, his travelling companion, on José Gaspar Rodriguez de Francia (1766–1840), the dictator of Paraguay, then an impenetrable country—a kind of North Korea of the time. Rengger’s testimony included second-hand information on the whereabouts of Aimé Bonpland (1773–1858), Humboldt’s collaborator, who also was forcibly kept in the country for over ten years (1821–1831) (Rengger and Longchamp 1827a, b, c).

Rengger took up geology and mineralogy late in life as a form of recreation. He relied on dedicated friends to pursue his interests, the young and energetic Peter Merian (1795–1883), professor at the University of Basel among others. In 1821, Merian had published the first volume of his own *Beyträge zur Geognosie,* which might have given Rengger the idea for his own collection: the introductory chapter to the latter’s 1824 work was composed one year later, in 1822. Like his friend Escher, Rengger wrote in German but was very much attached to French political and scientific culture and occasionally wrote in French. He was a typical member of the European cultivated élites leaning towards forms of moderate liberalism. Rengger feared aristocratic despotism as well as democratic mob rule, was active in educational reform, supported Johann Heinrich Pestalozzi’s (1746–1827) pedagogical innovations, and advocated state intervention to improve the conditions of the lower classes.

Rengger’s 1824 *Beyträge* elicited almost no reaction except a short review by Boué, who failed to mention Rengger’s reference to Lamarck. This was a particularly strange omission by an author who sympathized with a broad transformist view of the history of life on Earth. Boué singled out for comment the fourth article of the collection, on catastrophes seen as supporting Mosaic geology. Rengger, in the words of Boué, maintained that “the Holy Writs were not intended to teach us the structure of the Earth’s crust.” This was a point Boué himself insisted on throughout his career (Boué 1825, p. 178).

### 1827. “On the Changes which Life has Experienced on the Globe”

Jenkins’s attribution of the 1827 article to Boué has found powerful support in the sophisticated stylometric research published by Tanghe and Kestemont (2018). The 1827 article was overwhelmingly assigned to Boué by the computer analysis of a considerable number of texts, some signed and some anonymous; the 1826 article was comfortably attributed to Jameson. Though I am utterly incompetent to comment on stylometry, in private discussion with Koen Tanghe on the true authorship of the 1827 article, I surmised that several conceivable explanations are possible. For instance, the articles in English signed by Boué and the anonymous 1827 text might be found to be authored by the same individual (not Jameson) because they had been translated or edited by the same person. As we have already seen, it appears that all new solutions open up a host of intricate issues, which only archival research in Edinburgh will ultimately settle. In particular, as I will argue below, a better understanding of the editorial machinery at the *Edinburgh New Philosophical Journal* would be vital in establishing who assisted Jameson in his work, how issues were put together, and how the flow of information from European periodicals was dealt with. Indeed, the 1827 article, as well as the other two under discussion, was the product of the daily practices of borrowings and translations that traditionally characterized the life of periodicals.

Finding the original source for the 1827 “On the Changes which Life has Experienced on the Globe” (Anon. 1827a) was relatively easy: a matter of days rather than years, thanks to the strategy of translating back strings of words in the languages in which the original text was probably written. As I expected, this article too was a translation, this time from the *Bulletin des sciences naturelles et de géologie* edited and initially owned by Baron André-Étienne-Just-Pascal-Joseph-François d'Audebard de Férussac (1786–1836). The baron was also the author of the article, a review of the first set of instalments of the 1826 *Recherches sur les ossemens fossiles du département du Puy-de-Dôme* by Auguste Bravard (1803–1861), the Abbé Jean-Baptiste Croizet (1787–1859), and Antoine Claude Gabriel Jobert (1797c–1855) (Férussac 1827).^13^ Bravard and Jobert were well known to Férussac and collaborated in the *Bulletin*. The baron took their side against a rival publication and made use of the occasion to sum up and publicize his own key theoretical standpoints. As Irina Podgorny has magisterially shown, the dispute over the book unleashed deeply rooted personal and political hatreds, adding spice to the dormant life of Clermont Ferrand (Podgorny 2020).

Established in 1823 under the title *Bulletin général et universel des annonces et des nouvelles scientifiques*, in 1824, the publication was split up into several sections, eight in total, producing 170 volumes before its financial collapse in late 1831 (Férussac 1824; Taton 1947; Martin 2008). The two sections devoted to natural history and geology and to geography, the subjects Férussac was chiefly interested in, published the largest number of volumes. Férussac had involved key personalities of the Restauration in support of his venture. Grandees of the ultra-royalist party in power sat on the board of the Society he set up in 1827 to face the increasing difficulties encountered by the mammoth project. Some even lent considerable sums of money and helped raise further loans. The Dauphin (until 1824 Duc d’Angoulême) was asked to act as patron to the section devoted to medical sciences and eventually bought a majority share, prompting the resignation of two editors, François-Vincent Raspail (1794–1878) and Jacques Frédéric Saigey (1797–1871).^14^ It is highly possible that Férussac’s close proximity to key figures of the ultra-royalist establishment and the identification of the *Bulletin* with the Dauphin were held against him after the July 1830 Revolution, when Férussac failed to honour debts and his project for an international network of supporting sister Societies fell flat.

Almost fulfilling the dream of Samuel Hartlib’s Office of Address, Férussac set up a complex machine of European and transatlantic contacts, with the goal of obtaining complimentary subscriptions to periodicals and proceedings of scientific societies in exchange for one or all of the sections of the *Bulletin*. He actively promoted his plan by taking part, for instance, in the annual meetings of the Gesellschaft Deutscher Naturforscher und Ärzte (Heidelberg, September 1829), and in 1828, he published in English a pamphlet promoting the Company to attract shareholders from the United Kingdom (Férussac 1829, 1828c). At the height of its activity, the Parisian central office of the *Bulletin* claimed to receive almost 600 titles from countries in Europe, North and South America, and colonial capitals. The office of the *Bulletin* was regularly visited by scholars and cultivated amateurs eager to peruse the largest collection of current periodicals available in the country and, probably, on the whole Continent.

As I argue below, the presence of the *Bulletin* in Edinburgh is something to be expected. Férussac often summarized the contents of articles from the *Edinburgh New Philosophical Journal* including accounts of the meetings of the Wernerian Society. The very active publisher of the Edinburgh Journal Adam Black (1784–1874), had clearly entered into an exchange agreement with Férussac. Before dealing with the contents of the 1827 article lifted from the *Bulletin des sciences naturelles et de géologie*, however, we need briefly to consider the ways in which the original versions of the three articles under discussion reached Edinburgh.

## Travelling Texts

### The* Beyträge zur Geognosie* from Aarau to Edinburgh

In this short section, accounting, as far as possible, for the arrival in Edinburgh of the three articles under consideration, I will follow the chronological order of publication in the *Edinburgh New Philosophical Journal*. Let us first consider the more problematic case, the coming to Edinburgh of Rengger’s 1824 *Beyträge zur Geognosie.* Ami Boué is seemingly a good candidate for the role of go-between. He knew Jameson well. A close collaborator of Férussac, he was an expert in monitoring scientific literature throughout the Continent. As Archibald Geikie testified, Boué kept a filing system recording all geological publications and their contents he had read: this helps in understanding the ease with which he contributed endless entries to the *Bulletin* (Geikie 1881, p. 111). Although Boué privileged extensive field work and insisted on the importance of accurate observations, he had his own theoretical or “philosophical” agenda, as he would have put it, that included transformism.

Too many questions nevertheless remain unanswered, putting in doubt Boué’s role as intermediary in this particular instance. Why did he fail to take advantage of his role as a key collaborator of the *Bulletin* and avoid promoting Rengger’s work? As we have seen, his review of the book was short, ended on a critical note (Boué expressed surprise at serious omissions in the work), and only praised his Swiss colleague’s stand on the subject of Mosaic geology. Why did he refrain from commenting on the broad geohistorical issues discussed in the opening chapter? In the April issue of the 1826 volume of the Edinburgh journal—that is, a few months before the publication of the translation from Rengger—Boué wrote about his own views on species succession and geological directionalism. At the end of the article, he mentioned colleagues who agreed with him: Férussac, Alexander von Humboldt, Leopold von Buch, Joseph Fourier, Alexander Crichton, and Charles Daubeny (Boué 1825, pp. 103–104).^15^ Why did he omit Rengger? Even more perplexing is a short paragraph printed in the last instalment of volume 10, 1827, of the *Bulletin*, announcing that on page 293 of the *New Edinburgh Philosophical Journal* 1826, an article had been published on the importance of geology (Anon. 1827b). Clearly, the anonymous *Bulletin* collaborator knew nothing of Rengger and the *Beyträge zur Geognosie*: to him, the article in the Edinburgh journal was a novelty worth a brief notice, though the reference to Lamarck and the question of the succession of species left the writer totally indifferent and were not mentioned. Untypically, the instalment contained no contribution by Boué, only an anonymous notice of a memoir the geologist had published in October 1826 in the *Annales des sciences naturelles*, suggesting that perhaps Boué had not seen the instalment before press. Ironically, he might even have been visiting Rengger in Aarau when the instalment was put together (Boué 1879).^16^ Rengger was mentioned again, almost in passing on a few occasions, in later issues of the *Bulletin*, but no allusion was ever made to the views put forward in the essay colleagues at the *Edinburgh New Philosophical Journal* had translated, albeit without acknowledging the source.^17^

Among the foreign pupils of Jameson, I mentioned above Auguste Verdeil, a good friend and fellow student of Boué in Edinburgh. His father, a doctor like himself, played a significant role in the political and cultural life of Lausanne, as did he, after he went to live there in 1822, the year in which Rengger drafted his essay on the importance of geology. In 1799, Verdeil senior had been appointed chief health officer of the Swiss army, thus joining the top layer of the Swiss administration in which Rengger was a prominent personality. In later years, Verdeil senior was among the founders of the Société des sciences physiques de Lausanne (1819). Like his father, Auguste was very active in municipal health, philanthropic, and educational activities; in 1833, he became a member of the Municipal Council. Although we lack direct evidence, it is highly possible that Rengger knew the two Verdeils. Finally, the fact that Auguste was an admirer of Jameson and had a personal interest in geology would have made the connection with Rengger quite natural. Early in 1825, Verdeil had visited the United Kingdom, though in the admiring account of the system for road surfacing invented by John L. McAdam (1756–1836) he published back home, he only mentioned two towns, Bristol and London, not Edinburgh. It is not far-fetched to suggest—without direct evidence, needless to say—that Verdeil brought with him a copy of the opening essay of the *Beyträge zur Geognosie,* which he forwarded to Jameson, as coming from a prominent Swiss politician who had become a zealous supporter of geology—Wernerian geology, at that. Yet why did the journal’s editor decide to publish Rengger’s socially prestigious endorsement of geology and progressionism anonymously (Verdeil 1825)?

As far as Boué was concerned, he, and the *Bulletin des sciences naturelles et de géologie* with him, failed to advertise the interest of Rengger’s work and appeared to ignore the source of the 1826 *Edinburgh New Philosophical Journal* article. Rengger’s directionalist interpretation of the history of the Earth and its inhabitants, and his endorsement of the view that the decline in temperature was the main cause for the succession of flora and fauna—a key tenet of Férussac’s—should have elicited comment: Férussac was extremely keen to print in the *Bulletin* all opinions that could be reconciled with his own doctrines. Even in later years, when Boué could have listed Rengger among the geologists who doubted the constancy of species, he kept silent. As suggested above, the only hope for a final answer to the problem of how the *Beyträge* reached Edinburgh lies in further archival research on the daily working of the journal and the European network of correspondents Jameson entertained. It would indeed be of great interest to establish who were the collaborators Jameson relied on for translations or the perusal of foreign publications. Bill Jenkins has, for instance, established that William MacGillivray (1796–1852), an assistant to Jameson from 1823, translated sections from Lamarck’s *Histoire naturelle des animaux sans vertèbres*, preserved in manuscript form among the papers of his employer: did he also help with the journal (Jenkins 2019, p. 123)?

### Férussac’s *Bulletin* in Edinburgh

As already suggested, all available evidence indicates that an exchange of issues was in place between the *Bulletin des sciences naturelles et de géologie* and the *Edinburgh New Philosophical Journal*. The French periodical usually published short notices, ranging from a few lines to half a page, though Férussac reserved more space for topics he was particularly interested in, the discussion of his own works, and tenets in particular. The standard format of the *Bulletin*’s entries could have been useful to the Scottish journal only for the section “Scientific Intelligence” at the end of each issue: longer articles were not that common. Thus, there appears to have been no major borrowing by the Edinburgh journal from the French publication. One has the impression that in the exchange, it was the *Edinburgh New Philosophical Journal* that got the best part of the deal, having a number of its articles immediately publicized throughout the global reach of the *Bulletin*’s network. Still, the issues of the *Bulletin*, containing scores of short notices of recent publications, were precious to any journal editor, allowing them to form an idea of what was going on in European and extra-European scientific communities, without incurring the exorbitant (and impossible) cost of having to subscribe to hundreds of journals. In Edinburgh as elsewhere, the issues of the *Bulletin* were perused as a matter of routine. When the publication ceased late in 1831, many felt its loss. As Thierry Hoquet has shown, Baron Benjamin Delessert (1773–1847) financially assisted the establishment in 1833 of the *Archives de botanique*, to overcome the loss of the *Bulletin* (Hoquet 2007). The *Archives* were in the care of Jean-Baptiste-Antoine Guillemin (1796–1842), who had been the editor of the botany section of Férussac’s *Bulletin*. Nerée Boubée (1806–1862) launched his *Echo du monde savant* (1834–1846) in order to replace, in part, the *Bulletin*. In 1836, Cuvier’s former assistant, Charles Leopold Laurillard (1783–1853), incited the Parisian Société d’histoire naturelle to fill the gap by taking over the collection and distribution of information concerning new publications in the natural sciences, at least the books.^18^

Unlike the Scottish publication, the *Bulletin* always acknowledged the source of the notices it published, a practice consistent with the service the editors offered their readers and the journals that accepted the conditions of exchange. In other words, the *Bulletin* did not aim to be seen as original: its strength rested on the number and quality of the foreign publications it received, excerpted, and advertised. Still, Jameson’s journal translated two articles by Férussac, “On the Changes which Life has Experienced on the Globe,” in 1827, and, one year later, “Defence of Christianity, or Conferences on Religion … Moses Considered as a Historian of the Early Ages t. ii, p. 40,” which I will briefly consider below (Férussac 1827a, 1828a).

### The *Globe* in the Daily Working of the Edinburgh Journal

As already stated, the *Globe* was a major opposition journal in the tense 1820s, the years of ultra-royalist domination in France, although it was forced by the legislation on the press to limit its action to the cultural sphere. From the earliest issues, thanks to the work of Bertrand, the *Globe* offered timely coverage of sessions held at the Académie des sciences and the Académie de médecine. It published three issues a week, limited to 2 folios (4 numbered pages) each. This format cut the postal price almost to the level of single letters and ensured a relatively speedy delivery. The April 1, 1829 issue containing the article on Geoffroy reached Edinburgh fast enough to grant time for the translation to take place and for the volume to be put on sale on April 15 (Corsi 2016).

There was, of course, no lack of reporting on the activities of the Académie in the French periodical and daily press. For instance, the *Revue Encyclopédique* did a reasonable job at it, although its reports were rather short and laconic. Elections to membership were usually reported by the *Moniteur universel*. Yet, the *Globe*’s weekly pace, the promptness with which Bertrand did his job, and the at times lengthy summaries of memoirs read on the floor of the academy made the French publication very useful for a periodical like the Edinburgh journal. Jameson took full advantage of the service provided by the French publication. A systematic check, albeit limited to the *Globe* issues for 1828, has revealed that Bertrand’s summary of memoirs read at the Académie by, among others, Antoine Becquerel (1788–1878), Michel-Eugène Chevreul (1786–1889), Jean-Sébastien-Eugène Julia de Fontenelle (1780–1842), and Frédéric Villot were translated in the “Scientific intelligence” section of the Edinburgh journal. The entries were anonymous and did not indicate the original source of the texts (Becquerel 1828 and Anon. 1828; Chervreul 1828 and Anon. 1829a; Julia de Fontenelle 1828 and Anon. 1828; Villot 1828 and Anon. 1828). It will not come as a surprise that even the original memoir by Roulin on the changes incurred by European domesticated animals when transplanted to South America, which started the series of events leading to the 1829 memoir by Geoffroy, was duly covered (Roulin 1828 and Anon. 1828).^19^ Perusing *Le Globe* was therefore part of the routine at Jameson’s *Edinburgh New Philosophical Journal*.

## Ways of Reading

In this final section, I examine the three articles within the context of their authors’ known theoretical stands on the question of changes life might have incurred in the history of the Earth. In other words, I will move away from the historiographic issue of the “Edinburgh Lamarckians” and will attempt to read the texts, as far as possible, in their own terms, within their intellectual and social ecosystem. Obviously, readers then and now, were and are free to read into the articles overtones, innuendoes, or even theoretical choices their authors were far from sharing or were firmly opposed to. Equally obviously, it is still worth the effort to distinguish what historians have read into these articles and what their authors actually meant and seemingly wished to convey. Even the case of Bertrand’s 1829 account of the memoir by Geoffroy, when contrasted with what he personally thought about any form of transformism, reveals little-studied dynamics within Parisian radical circles of the Restauration, which were often indifferent to species transformation in general and to Lamarckism in particular.

### Albrecht Rengger and Progressionism

I have already indicated that the coming to Edinburgh of Rengger’s *Beyträge zur Geognosie* is difficult to trace. It is equally difficult to account for Rengger’s theoretical commitments, since we only have his short chapter to rely on. As he wrote to his friend Escher, the text was “a kind of introduction in which I try to present geognosy from a higher point of view than is usually considered; a kind of justification for its disciples.”^20^ It is significant that in his reply Escher made no objection concerning the protracted discussion of Lamarck’s doctrines and their application to geohistory: quite the opposite. Rather, Escher felt that the concluding remarks on the usefulness of geology were detracting from the high philosophical tone of the chapter.^21^

As far as I have been able to verify, Rengger only referred to Lamarck in this brief text. The bulk of his works, essentially articles, some of which were collected in the *Beyträge* and in the volume *Über den Umfang der Juraformation* (1829), consisted of reports of excursions and field observations. Still, the chapter offers a fascinating insight into the complex ways a member of the European cultivated elites read Lamarck’s work in the very early 1820s. Of the three articles under review, Rengger’s was the only one that openly insisted on the centrality of Lamarck to current debates on the succession of fauna and flora. The old politician had read the *Philosophie zoologique* very carefully, and several passages of his chapter echo similar passages in the work of the French naturalist. The reference to domestication as attesting the mutability of species was taken from a passage in Lamarck; indeed, Rengger was one of the few commentators to notice it (Anon. 1826, p. 298; Lamarck 1809, vol. 1, p. 229). Equally inspired by *Philosophie zoologique* were the passages discussing the Egyptian Ibis and the outline of the development of life from simple organisms to man.

Rengger did, however, feel that Lamarck had to be updated and corrected in important ways. He was aware that Lamarck had refused to rely on fossils as evidence of species change. According to the French naturalist, all forms of life that ever existed were documented in the tree of life. Taxonomy was the only reliable guide to the historical succession of organic configurations ascending from the simple to the complex, up to man, the most complex form of life. Species extinction was a rare event, usually the result of man’s action. Even the fossil shells he had so accurately described did not suggest historical anteriority but only ancient patterns of geographical distribution. Thus, a few marine invertebrates found alive in Australia were only met with as fossils in European formations. Lamarck was convinced that ammonites were still thriving in the depths of the oceans, and only when the beds of today’s seas will one day emerge as lands would we find their remains. Tellingly, in his *Histoire naturelle des animaux sans vertèbres* (1815–1822), Lamarck had classified fossil species together with the living ones.

Rengger suggested that Lamarck’s approach could valuably be applied to explain the history of life, clearly showing marked evidence of progress from less to more complex forms. When shorn of all flights of imagination (Rengger shared this rather common critique of the French naturalist), the Lamarckian doctrine helped to turn systems of classification into accounts of the historical succession of forms and solved crucial issues debated by naturalists: for instance, whether species were each allotted a lifespan, like individuals, or whether their survival or disappearance was linked to stable or to changing environmental conditions. Rengger was thus aware of the discussion on the lifespan of species heightened by the publication in 1814 of the *Conchigliologia fossile subappennina* by Giovan Battista Brocchi (1872–1826). As is well known, even Charles Lyell and the young Charles Darwin took Brocchi very seriously indeed (Corsi 1978). Rengger rejected the view that species, like individuals, were born and died at their appointed time and opposed the catastrophist interpretation of extinction. Alterations in environmental conditions were likely to weaken species, which “at length become perfectly extinct” (Anon. 1826, p. 298).

The 1826 article has been commented upon too many times to deserve further analysis. It is, however, important to insist on the ultimate religious overtones and assumptions present in the text. As Boué had pointed out when reviewing the *Beyträge zur Geognosie*, Rengger vindicated the independence of geological research from the biblical narrative of creation—which did not mean, however, that he believed the two accounts could ever be fundamentally at odds. In the chapter translated for the Edinburgh Journal Rengger called upon the important results obtained by geological research: “these facts concur with historical testimony, in representing the elevated platforms of Asia, as the cradle of the human race, and in explaining their diffusion from that centre; and the traditions of deluges, found among all the nations of antiquity, are corroborated by the still existing traces of those violent events” (Anon. 1826, p. 295). Although he was not inclined to catastrophism and considered that there had been several “deluges” within historical times rather than a single universal flood, Rengger did not agree with Cuvier that the remains of rhinoceroses and elephants found in the furthest northern region of Asia belonged to species adapted to the inhospitable environments where they had died: the remains “of the rhinoceros found on the shore of the Wilhui, and of the mammoth at the mouth of the Lena, are likewise indications of sudden changes in those places” (Anon. 1826, p. 299). Finally, organic progressionism and the succession of fauna and flora “must be found more worthy of its [nature’s] first Great Author than the limited conceptions we commonly entertain” (Anon. 1826, p. 297).

Nothing was further removed from Rengger’s intention than supporting a radical and potentially subversive scientific agenda. His chapter did not shock his friend Escher because there was nothing to be shocked by—Rengger was, after all, a trusted pillar of the Swiss establishment: a philanthropist and moderate reformer. The high philosophical ground he had chosen as the theoretical location for his assessment of the debates on species change and succession allowed him to ward off all association with materialist and radical leanings. To think otherwise would have amounted to doubting the word of a gentleman amateur whose only goal was the advancement of knowledge and of a philosophical approach to natural sciences in general and to geology in particular. At the time, Rengger was not the only member of the European political elites endorsing a broadly Lamarckian approach purged of all materialistic overtones: his colleague, the Belgian geologist and conservative Catholic politician Jean-Baptiste-Julien d'Omalius d'Halloy (1783–1875), was doing exactly the same. D'Omalius d'Halloy, like Rengger, would have been amazed at being suspected of radicalism because of his cautious endorsement of Lamarck, as would their common friend Ami Boué (De Bont 2007).

### The Enigmas of Férussac

As suggested above, the main argument of the short 1827 *Edinburgh New Philosophical Journal* article, extracted from a review by Férussac, was quite straightforward. The Earth at the beginning was much hotter than now, volcanoes were much more active, and a warm climate evenly extended over all its surface. Plants and animals were also more uniformly spread than indicated by today’s fractured biogeography. The central heat that Buffon had firstly hypothesised, and contemporaries such as Louis Cordier (1777–1861) and Fourier had confirmed, slowly dissipated with the slow passing of tens of centuries. As a consequence, life forms in search of warmer climates had moved from the polar regions towards the tropics and from the top of mountains to the plains, although a large number perished in the process. Organisms were always and irremediably adapted to their conditions of existence: climatic change entailed death. Catastrophes or universal deluges had no place in Ferussac’s geohistory. Events, physical and organic, always occurred at the local level—the gradual cooling had different effects in different locations, depending on latitude, height, proximity to oceans or internal seas, and so on. Since sea levels had also decreased, the surface of the earth was punctuated by a number of successive and alternating freshwater and sea water basins. Contemporary readers would easily have caught a reference to Cuvier’s and Alexandre Brongniart’s work on the Paris basin, a freshwater lake invaded on several occasions by the sea.

In his 1827 short text, Férussac refused to endorse a progressionist interpretation of the history of life on Earth. He believed that geothermic conditions had progressively favoured the establishment of milder climates, but he saw no trace of organic progress. Furthermore, since from the Tertiary era to today everything happened at the local level, in given areas or “basins” (the term borrowed from geography also included the organisms they supported), some forms of life lasted longer than in other areas before perishing under the pressure of changing circumstances:

Certain primitive types have indeed completely disappeared, but they are found existing at various epochs, and their remains are blended with those of modern times; along with new species of types still existing, we find some of anterior epochs; certain genera that yet obtain are common to all the terms of the series; and toward the end of the series, we find the remains of some of our present species along with ancient types and extinct species. (Anon. 1827a, p. 300)

This was a view with which Charles Lyell could have agreed.

Férussac’s reliance on physical laws slowly but inexorably shaping the surface of the earth, and his description of the “regular, general, and continued natural causes of the modifications which life has undergone,” has legitimately been interpreted as the expression of a form of a transformist worldview. Yet, the text does not authorize ranking Férussac amongst the “Lamarckians.” Lamarck had categorically excluded the legitimacy of a progressionist history of the earth, denied that sea levels had decreased, and never spoke of internal heat. He would have agreed, needless to say, that changes in the environment prompted organic change: a generic point many would have subscribed to, albeit with different overtones. A closer look at Férussac’s entire production reveals once again the complexity of theorizing on the successions of life forms during the 1810s and the 1820s. The brief discussion here will be limited to two points only: a text by Férussac commentators have considered as “semi-Lamarckian,” and why this reading is unwarranted.

Férussac accepted the invitation of a former comrade in the corps of the army general staff, Jean-Baptiste Bory de Saint-Vincent (1778–1846), to contribute an entry on the geographical distribution of “molluscs and shellfish” to the *Dictionnaire classique d’histoire naturelle* (1822–1831). The dictionary circulated widely among cultivated French and European readers. Férussac seized the opportunity to make his views known outside the circle of conchologists and academic audiences. He thus offered an overview of his theory on the successions of species. The conclusions to the entry provided an accurate synthesis of his thoughts:

1°. The analogy of stations and destination, that is, of the conditions of existence and of the role to fulfil, is the general law that has presided over the distribution of life on the globe; 2° the changes life has experienced at the surface of the globe have been gradual; life has not been renewed; races have not been modified, but in so far as the conditions of existence changed or new conditions were established, new species have replaced those that could no more exist and had no role to fulfil, until the time when an equilibrium was established among acting causes, for each portion of the surface in turn. (Férussac 1825, pp. 269–270)

In other words, Férussac denied that catastrophic mass extinctions, followed by the replacement of entire faunas and flora, had ever taken place. Yet, he equally denied that species had been modified. Férussac would have been surprised to be considered a Lamarckian: his opposition to any form of transformism was a constant feature of his work (Godlewska 1999; Blanloeil 1988).^22^ Early in his career, Férussac had crossed swords with the mining engineer Cyprien-Prosper Brard (1786–1838), a protégé of Barthélemy Faujas de Saint-Fond (1743–1817) and of Jean-Claude Delamétherie (1744–1821). The matter of contention was the possibility that marine and freshwater invertebrates could adapt to changes in the liquid they were plunged in. In polemical memoirs attacking Cuvier and Brongniart’s work on the Parisian basin, Brard denied that extinction was the necessary outcome of the transition of invertebrates from freshwater to sea water and vice versa. He had conducted experiments showing that freshwater organisms could adapt and survive in a liquid of increased salinity. Brard had been a pupil of Lamarck at the Muséum and acknowledged his allegiance to his teacher’s theories (Brard 1812; Corsi 1988, pp, 208–209).^23^ It is interesting to note that Férussac imputed Brard’s limited form of transformism to the latter’s reliance on doctrines put forward by Delamétherie and Faujas de Saint-Fond, not by Lamarck. Indeed, throughout his production Férussac never mentioned Lamarck in connection with discussions on the succession of life forms, engaging only, very respectfully, with the latter’s invertebrate taxonomy.

The comments on Brard were shaped by Cuvier’s methodological and scientific critique of transformism: “I will not imitate those who, having collected a few isolated facts, believe they can explain the great phenomena of nature. Following the example of the learned authors of the *Géographie minéralogique des environs de Paris*, I will abstain from venturing opinions further facts could contradict” (Férussac 1814, p. 74). With Cuvier, Férussac insisted that organisms were inextricably linked to the physical conditions they were living in. Life forms were an aggregate of tightly interconnected organs and functions, so that one part could not adapt without entailing changes in the entire structure: “one ought to suppose that their respiratory system, their habits, their nourishment had completely changed.” A terrestrial, freshwater or seawater invertebrate could only exist in the environment all its organs were adjusted to (Férussac 1814, p. 75).

Faithful to his anti-transformism, anti-catastrophism, and anti-directionalist history of life, Férussac abstained from moving one step ahead of the “facts” he had established. What he repeated over and over, the general “fact” he had proudly established, was that new forms of life appeared in circumscribed “basins,” areas from where they could not move, since they were adapted to the conditions of life in which they existed. True to his methodological caution, he repeatedly warned that he was mainly referring to Tertiary formations and to the organisms he knew best, molluscs and shellfish. He did not feel he could speak of earlier times or of different organisms.

It is noteworthy that Férussac avoided mentioning alternative explanations: the polemic against Brard’s form of transformism was a rare event. He did not refer to the theories put forward by his friend Bory de Saint-Vincent, who took advantage of his role as editor of the *Dictionnaire classique* to add, at the end of Férussac’s entry, a reference to his own article “Création.” According to Bory, aggregations of molecules of “active matter” (*matière agissante*) and of green matter (*matière verte*) generated simple organisms intermediary between plants and animals. From these primitive aggregates of molecules, higher forms of life evolved by molecular additions, thus explaining how and why even the most isolated island lost in the middle of the oceans could host autochthonous forms of life. He famously referred to the dodo, a now extinct creature only to be found (he thought) on the isle of Mauritius, as a product of the creative power of nature (Bory de Saint-Vincent 1824). Again, faithful to his methodological guidelines, Férussac abstained from commenting or even mentioning the doctrines proposed by colleagues he admired, like Lamarck, or collaborated with, like Bory.

Férussac carefully avoided multiplying the instances of the introduction of new species at the surface of the Earth. The organisms he was relying on, molluscs and shellfish, displayed a marked form of dimorphism and a general tendency to variation. Naturalists, he continued, were often inclined to consider such variations (displayed by both fossil and living forms) as true species, thereby multiplying the instances of organisms specific to given basins. Varieties of the same species found at great spatial or historical distances could easily albeit wrongly be taken as isolated true species, if naturalists failed to consider intermediary forms in the repertoire of variations displayed by a specific organism.

As stated above, Férussac was on good terms with influential representatives of the ultra-royalist party, up to the Dauphin, whom he courted. He was particularly pleased to express his full agreement with Denis Frayssinous’s (1765–1841) *Défence du Christianisme* (1825) by reviewing favourably the section of the book the prelate devoted to geology and the Mosaic account of creation (Frayssinous 1825, vol. 2, pp. 36–71; Frayssinous 1836, vol. 2, pp. 38–75). Appointed Bishop of Hermopolis *ex partibus infidelium* (an honorary position) in 1822, from 1824 to 1828 Frayssinous was the powerful Minister of Ecclesiastical Affairs and of Education (Roquette 2007). He was well known for his militant anti-materialist and ultra-royalist stand. In November 1822, as Chancellor of Paris University, he (in)famously closed down the Faculty of Medicine and fired eleven prominent professors there, including the venerated Philippe Pinel (1745–1826), whose death a few months later was attributed to the anguish caused by the measure.

As already documented, Férussac’s review of Frayssinous was translated in the *Edinburgh New Philosophical Journal* (Férussac 1828a). The conchologist ably exploited Frayssinous’s invitation to geologists to carry on their work without fear of interference from biblical scholars. The Bishop relied on Saint Augustin (among others) to argue that the language of Genesis was often poetical: the “days” of creation did not necessarily mean period of twenty-four hours. Férussac and Geoffroy, who also praised Frayssinous, were pleased to refer to the *Défence du Christianisme* to fend off criticisms from extreme Catholics and royalists arguing that geologists were treading on dangerous grounds. Lyell himself, in his review of George Poulett Scrope’s *Memoir on the Geology of Central France*, quoted the *Bulletin* and praised both Frayssinous and Férussac for their stand on the relationship between geology and the Holy Writings (Lyell 1827, pp. 481–482).

Commentators who have considered Férussac a fellow-traveller of Lamarck or have legitimately interpreted detached passages of the 1827 article as an endorsement of transformism have been misled by historiographic assumptions overlooking the complexity of the natural history scene of the 1810s and the 1820s. In spite of Charles Lyell’s August 1823 letter to his father dismissing Férussac as a theory monger and a cabinet naturalist, the solution to the succession of fauna and flora proposed by the French conchologist was, in part at least, in line with the one Lyell was to announce a few years later—no catastrophes or mass extinctions but a piecemeal loss of species, followed by the equally piecemeal appearance of new species fulfilling the same role in a given environment.^24^ On reading Férussac, Frayssinous would have seen a further praiseworthy example of the theoretical prudence advocated by Cuvier, to whose judgement both the ecclesiastic and the conchologist were adhering. After all, the Dauphin himself appeared to approve Férussac’s work and supported his publishing ventures. Nobody dreamt of imputing radical leanings to Férussac’s discussion of the succession of flora and fauna, and many would have praised his refusal to speculate on such a momentous matter.

When endorsing the moderate version of Mosaic geology deployed by Frayssinous, Férussac was not being diplomatic or opportunistic. For instance, when the Bishop explained that according to Genesis the early vegetables prospered even without the sun, created a “day” later, Férussac informed readers that a British author, the (to us) almost unknown Alexander Crichton (1763–1845), had argued that the primitive vegetation grew thanks to the internal heat of the planet, not to the action of the sun—a further confirmation that independent geological research produced results compatible with a judicious reading of Genesis (Crichton 1825).^25^

The 1827 short article by Férussac has inevitably called for the longest comment. As is clear by now, reading a text out of its proper context makes historiographical assumptions dominate our reading of it. Trying to listen to voices almost blurred by our own assumptions is probably the biggest challenge in making sense of the Edinburgh “Lamarckians” in general and of Férussac in particular.

### The Paradox of Alexandre Bertrand

As already shown, reports on the activities of Geoffroy were a constant feature of the science section of *Le Globe*. The fact that Roulin was a long-time friend of both Leroux and Bertrand, and a relative of Bertrand at that, made the science editor particularly zealous in reporting the debates aroused by the memoir his brother-in-law, still an unknown travelling naturalist, had read to the Académie at the end of September 1828. After the death of Bertrand in 1831, it was Roulin who took his place as the science editor at *Le Globe* for the last months in the life of the journal (Viard 1986, p. 145). Roulin then moved on to write science reports for the daily *Le Temps* (1829–1842) and helped Arago in setting up the *Comptes rendus de l’Académie des sciences* (1835); until his death, Roulin single handedly compiled the summaries of papers read, and of debates held, on the floor of the Académie.

The case of the 1829 article is markedly different from the ones discussed above. Bertrand was only doing his job as science editor, reporting on doctrines formulated by third parties. It is, however, interesting to point out that, in spite of the accurate summary he provided of the memoir Geoffroy read at the Académie on March 23, 1829, Bertrand had mixed feelings—to say the least—concerning transformism and the possibility that living organisms were the descendants of fossil ones. In 1824, Bertrand published a volume of *Lettres sur les révolutions du globe* intended for the general public eager to know more about fashionable scientific topics (Bertrand 1824). It is a very elementary work, a kind of anthology of annotated extracts from the works of a handful of contemporary geologists, Cuvier in particular. The considerable and lasting success of the book prompted the publication of new revised editions from 1826 to 1879; its sale helped to supplement the meagre resources of Bertrand’s widow. As a marketing strategy, successive editors asked famous scientists to add a note of comment or a short chapter, though no significant change was introduced in the main text. For the fifth edition, published in 1839, François Arago, Élie de Beaumont, and Alexandre Brongniart were asked to add their names to the rostrum of advisers. For the sixth edition (1865), edited by Bertrand’s son Joseph (1822–1900), by then a famous and powerful member of the Académie, several geologists were enlisted (Charles and Henry Sainte-Claire Deville, Achille Delesse).

For the third edition he personally supervised (1828), Bertrand had obtained from Adolphe Brongniart a preprint copy of the first two instalments of the latter’s work on fossil vegetables and summarized the section on the warmer temperature of former ages (Brongniart 1828a, b, 1837). Bertrand did not appear to be familiar with Brongniart’s *Prodromus*, also published in 1828, or the latter’s memoir published in the *Annales des sciences naturelles* of November 1828 (Brongniart 1828a, b). In the memoir, the botanist had put forward his view that the warmer temperature was accompanied by a much higher presence of carbon dioxide in the atmosphere of the early Carboniferous. This explained the momentous quantity and dimensions of the rather primitive flora of the period and the fact that dead plants were better preserved from decomposition, thus facilitating their transformation into coal. More complex plants and warm-blooded animals requiring significant oxygen intake for their survival started developing only when the flora of previous ages had purified the atmosphere and captured enormous quantities of carbon. Brongniart’s work was greeted with great favour by contemporaries, and particularly so by Férussac and Geoffroy. Geoffroy made use of Brongniart’s work in his 1829 text summarized by Bertrand, although he typically avoided acknowledging the source. The 1828 edition of Bertrand’s popular work helped to publicize Brongniart’s conclusions on geoclimatic directionalism and its impact on successive fauna and flora. It is highly possible that Geoffroy’s first introduction to the views put forward by Brongniart was through Bertrand’s compilation.

Brongniart’s memoir was immediately translated in the *Edinburgh New Philosophical Journal* (Brongniart 1829). In a very rare editorial comment, Jameson appended a note to the first page of the translation, stating that the article offered views “similar to those we have been in the practice of delivering to our pupils in the University, both in the class-room and during our geological excursions.” It is important to stress that Brongniart did not endorse the interpretation of his findings suggested by Geoffroy and never expressed sympathy for transformism. Equally, Jameson’s reference to Brongniart’s form of progressionism cannot be automatically taken as approval of the brand of transformism Geoffroy put forward in his 1829 memoir. As we have seen, Férussac, Bertrand, and Brongniart himself denied that climatic change brought about the modification of species.

In spite of his well-established connection with the political and cultural radicalism of the 1820s, like Jameson Bertrand remained a faithful follower of Cuvier. To the dismay of Férussac, the very title of his work, *Lettres sur les révolutions du globe*, suggested that Bertrand favoured a catastrophist interpretation of the history of the Earth (Férussac 1828b). Concerning transformism, Bertrand reproduced the section from Cuvier’s *Discours sur les revolutions de la surface du globe* where the famous naturalist pretended that Lamarck and Delamétherie shared identical views, a rehash of the vagaries of Benoît de Maillet’s (1656–1738) *Telliamed* and of the German Johann Christian Rödig (1772–1863).

Bertrand had conscientiously done his work as science editor of the *Globe*. He reported as faithfully as he could the contents of the memoir read by Geoffroy. It is also possible, but we lack solid evidence for this, that Geoffroy had given a copy of his text to Bertrand. Geoffroy was known to court men of letters working for periodicals and encyclopaedias, and he was rather successful at that. Increasingly isolated within the Académie, in the early 1830s his reputation grew thanks to sympathetic reviewing from the press and extensive coverage by leading encyclopedias (Corsi 2011). As is often the case within intellectual circles, from September 1828 to March 1829, Geoffroy, Leroux, Roulin, and Bertrand engaged in an exchange of favors. Geoffroy’s 1829 memoir, also thanks to Bertrand and the *Edinburgh New Philosophical Journal*, quickly attracted universal attention.

## Conclusion

The aim of this contribution has been to identify the authors of three anonymous articles published in the *Edinburgh New Philosophical Journal* from 1826 to 1829 dealing with the succession of fauna and flora throughout the ages of the Earth. I have also tried to establish the channels of communication that brought the three texts to the attention of the editor of the Edinburgh journal. Finally, I have attempted to read the texts, as far as possible, from the point of view of their authors. It has by now become abundantly clear that the question of Lamarckism, legitimate as it is, has to some extent distorted the reading of the articles. To the upper-class old politician Rengger, Lamarck was surely a reference point, and a selective reading of the work of the French naturalist had become part of the author’s “philosophical” reflections on geology and its standing in contemporary culture. The former soldier, conchologist, and publishing entrepreneur Férussac never mentioned Lamarck in connection with the question of species, opposed transformism, and coupled a progressionist history of life with a directionalist Earth history. The radical doctor and “man of letters” Bertrand shared Cuvier’s disdain for Lamarck and limited his task to reporting as faithfully as possible on what Geoffroy had said on the floor of the Académie. Yet, he was not convinced by the conclusions Geoffroy had reached.

As I have repeatedly pointed out above, much work remains to be done concerning the editorial practices at the Edinburgh journal, the network of exchanges of texts and information that made the life of the periodical—indeed, of all periodicals—possible, and the assistants Jameson relied on in his daily work. A systematic study of all the issues of the Edinburgh journal will reveal the extent to which translations played a significant role in each instalment. More importantly, analysis of the range of topics the borrowings covered will help highlight the editorial strategy and the scientific agenda of the journal which, needless to say, changed over time. The second half of the 1820s calls for special attention, in the British Isles as well as in France, in Edinburgh as well as in Paris. For complex social and political reasons, representatives of wide-ranging cultural constituencies—in literature, philosophy, science, theology—appeared interested in exploring territories which the political reaction following 1815 and the renewed alliance between the Altar and the Throne had declared subversive and therefore forbidden. The attempt by several leading contributors to *Le Globe* to find a reforming middle ground between repression and rebellion, absolute monarchy and democratic temptations, attracted attention. In the natural sciences, Cuvier’s indictment of German idealistic morphology, the doctrine of the unity of type or of embryonic recapitulation, and of the various theories of species modifications discussed at the time, generated a backlash (Cuvier 1825; Royer-Collard 1828). Debates on the succession of species took on a kind of speculative urgency that should not be confused with acritical adhesion to earlier doctrines, Lamarck’s in particular. It is not surprising that the possibility of explaining the succession of fauna and flora in terms of natural laws—for many, providentially ordained by the Creator or resulting from the repeated interventions of the First Cause as part of a design conceived from the beginning—elicited the attention of representatives of conservative as well as moderate members of the elites (Corsi 1988, 2009, 2011).

The three articles here reviewed strongly suggest widening the geographical as well as the theoretical scope of investigations into the debates on species change during the early decades of the nineteenth century. The vitality of the European cultural space and the strength of the networks of exchange and communication at the Continental level render concentration on a single country, or a single town (in our case Edinburgh), potentially counterproductive. Similarly, reducing all debates on species change to debates on Lamarck and his controversial heritage amounts to ignoring the many strategies contemporaries had elaborated to make sense of the succession of fauna and flora throughout the history of the Earth.

## Footnotes

1 Secord (1991) also reconstructed the long history of the attribution to Grant, first put forward by Loren Eiseley in his *Darwin's Century: Evolution and the Men Who Discovered It*, London: Gollancz, 1959. For a full account of attributions, see Tanghe and Kestemont (2018) and Jenkins (2019).

2 Janet Browne (1995, p. 554, n. 37) also expressed reservations on the attribution to Grant.

3 Jenkins 2019; see Corsi 2011 for a discussion of Geoffroy’s shifting and often opportunistic attitude towards Lamarck. During the early 1830s, Geoffroy adopted Cuvier’s disparaging tone towards Lamarck.

4 Secord (1991, pp. 4–5). Secord documented how the new preface, dated November 25, 1826, opened with a defense of geology that showed similarity with analogous arguments deployed in the 1826 anonymous essay.

5 *Doctrinaires* indicate Pierre-Paul Royer Collard (1763–1845) and his group of constitutional monarchists, whereas *Eclectics* indicate the followers of Victor Cousin (1792–1867), later Minister of Education. After the July 1830 Revolution, the editorial team split on political grounds.

6 On Leroux and his relationship with Geoffroy and the latter’s family, see Corsi (2011).

7 “Quiconque connaît les écrits ou a entendu les leçons de M. Geoffroy devait s’attendre à des idées hardies, originales, exprimées dans un style presque toujours fort et pittoresque, mais entaché de tournures bizarres, d’expressions incorrectes et d’une sorte d’emphase qu’on lui pardonne volontiers.”

8 Don Antonio Zea, ambassador of Colombia to Paris, had been charged by Simon Bolivar to recruit naturalists to help establishing scientific institutions in the new Republic and to prospect natural resources.

9 Roulin married Manette and Bertrand Caroline, daughters of Joseph Blin (1764–1834), a federalist politician of Rennes.

10 “Je venais de lire, dit-il, d’importantes observations communiquées à l’Académie par M. le docteur Roulin: l’esprit préoccupé d’anciennes méditations sur les animaux antédiluviens, il m’échappa, en terminant mon rapport, une réflexion qui, pour être bien comprise, aurait nécessité de plus grands développements; on le fit remarquer, et on voulut bien insister pour que je me chargeasse de les donner.”

11 Boué’s autobiography, clearly the product of senile vanity, listed all the great minds he had met during his impressive life.

12 I wish to acknowledge and thank Prof. Tanghe, at the University of Ghent, for his generous support and the endless good-humored mail exchanges. On his involvement in the Edinburgh Lamarckians debate, see below.

13 See Anon.(1827a): the Edinburgh journal translated pp. 92–95.

14 Férussac (1827c). The title page of the volume boasted that the *Bulletin* was put “Sous les auspices de Monseigneur le Dauphin par la Sociètè pour la propagation des connaissances scientifiques et industrielles.” Férussac listed all the difficulties he was facing to continue his project, at great financial and personal cost (Martin 2005). Csiszar has established the sale of a majority of shares to the Dauphin (2018, p. 100).

15 The last section of the article was heavily indebted to works by Férussac, discussed below.

16 During Spring 1827, Boué spent time traveling and paid a visit to Rengger, “le vieux patriote.”.

17 See, for instance, Rengger (1831), mentioned in *Bulletin de sciences naturelles et de géologie*, 26 (1831), p. 138.

18 Silvia F. de M. Figueirôa kindly allowed me to see her forthcoming article, “Nérée Boubée (1806–1862) and l’*Écho du monde savant*: Activism for Science Popularization and Communication in Nineteenth-century France.” C. L. Laurillard to G. L. Duvernoy, 22 February 1836, in Cordot (2010, p. 296).

19 It would be interesting to submit these shorter entries to stylometric analysis to see whether the program may help identify a common translator.

20 Rengger to Escher, 22 February 1822, in Wydler (1847, vol. 2, p. 307), translated by Koen Tanghe.

21 Escher to Rengger, 8 May 1822, in Wydler (1847, vol. 2, p. 308).

22 Godlewska (1999, p. 170) defined the passage we have quoted as a “very carefully worded semi-Lamarckian conclusion.” Blanloeil (1988) argued for the life-long anti-transformism of Férussac.

23 Brard (1812, p. 257): “I admit these gradual changes, I subscribe to Messrs. Delamétherie and Lamarck’s systems.”.

24 Lyell to his father, Paris, August 10, 1823 in Lyell 1881, vol. 1, pp. 139–140. Lyell wrongly accused Férussac of being a cabinet naturalist without field experience—as a military man, the French naturalist had visited locations in, and collected specimens from, Spain, Belgium, the German states, and Switzerland. Lyell also accused Férussac of being a builder of geological systems—compared to many of his contemporaries in France and elsewhere in Europe, the French conchologist could be considered as a rather restrained theorizer.

25 Férussac (1828a, p. 87) hinted that Crichton had merely “extended” his own work.
